# Metabolic Profiles of Pregnancy With Polycystic Ovary Syndrome: Insights into Maternal-Fetal Metabolic Communication

**DOI:** 10.1210/clinem/dgaf057

**Published:** 2025-01-29

**Authors:** Huisheng Ge, Dongni Huang, Lunbo Tan, Dan Luo, Liu Zhou, Hong Liu, Yilan Zhang, Dandan Liu, Xixi Wu, Lulu Wang, Liling Xiong, Yang Yang, Ting-Li Han, Chengjin He, Hongbo Qi

**Affiliations:** Department of Obstetrics and Gynecology, Women and Children's Hospital of Chongqing Medical University (Chongqing Health Center for Women and Children), Chongqing 401147, China; Chongqing Key Laboratory of Maternal and Fetal Medicine, Chongqing Medical University, Chongqing 400016, China; Department of Obstetrics and Gynecology, Chengdu Women's and Children's Central Hospital, School of Medicine, University of Electronic Science and Technology of China, Chengdu 610015, China; Department of Obstetrics and Gynecology, Women and Children's Hospital of Chongqing Medical University (Chongqing Health Center for Women and Children), Chongqing 401147, China; Chongqing Key Laboratory of Maternal and Fetal Medicine, Chongqing Medical University, Chongqing 400016, China; Department of Obstetrics and Gynecology, Women and Children's Hospital of Chongqing Medical University (Chongqing Health Center for Women and Children), Chongqing 401147, China; Chongqing Key Laboratory of Maternal and Fetal Medicine, Chongqing Medical University, Chongqing 400016, China; Department of Obstetrics and Gynecology, Chengdu Women's and Children's Central Hospital, School of Medicine, University of Electronic Science and Technology of China, Chengdu 610015, China; Department of Obstetrics and Gynecology, Chengdu Women's and Children's Central Hospital, School of Medicine, University of Electronic Science and Technology of China, Chengdu 610015, China; Department of Obstetrics and Gynecology, Chengdu Women's and Children's Central Hospital, School of Medicine, University of Electronic Science and Technology of China, Chengdu 610015, China; Department of Obstetrics and Gynecology, Chengdu Women's and Children's Central Hospital, School of Medicine, University of Electronic Science and Technology of China, Chengdu 610015, China; Department of Obstetrics and Gynecology, Chengdu Women's and Children's Central Hospital, School of Medicine, University of Electronic Science and Technology of China, Chengdu 610015, China; Department of Obstetrics and Gynecology, Chengdu Women's and Children's Central Hospital, School of Medicine, University of Electronic Science and Technology of China, Chengdu 610015, China; Department of Obstetrics and Gynecology, Chengdu Women's and Children's Central Hospital, School of Medicine, University of Electronic Science and Technology of China, Chengdu 610015, China; Department of Obstetrics and Gynecology, Chengdu Women's and Children's Central Hospital, School of Medicine, University of Electronic Science and Technology of China, Chengdu 610015, China; Department of Obstetrics, The First Affiliated Hospital of Chongqing Medical University, Chongqing 400016, China; Department of Obstetrics and Gynecology, The Second Affiliated Hospital of Chongqing Medical University, Chongqing 400010, China; Joint International Research Laboratory of Reproduction and Development of Chinese Ministry of Education, Chongqing Medical University, Chongqing 400016, China; Department of Obstetrics, The First Affiliated Hospital of Chongqing Medical University, Chongqing 400016, China; Department of Obstetrics and Gynecology, Women and Children's Hospital of Chongqing Medical University (Chongqing Health Center for Women and Children), Chongqing 401147, China; Chongqing Key Laboratory of Maternal and Fetal Medicine, Chongqing Medical University, Chongqing 400016, China

**Keywords:** polycystic ovary syndrome, metabolic communication, umbilical cord serum, placenta

## Abstract

**Context:**

Polycystic ovary syndrome (PCOS) pregnancies are linked to metabolic disorders affecting maternal and fetal outcomes, with maternal metabolites differing from those in normal pregnancies.

**Objective:**

To investigate the metabolic communication at the maternal-fetal interface in PCOS pregnancies.

**Design:**

Placenta and umbilical cord serum were analyzed using gas chromatography-mass spectrometry. In-depth analysis was performed with clinical characteristics.

**Setting:**

Placenta and umbilical cord serum were analyzed using gas chromatography-mass spectrometry, alongside clinical characteristics.

**Participants:**

Forty-five uncomplicated PCOS pregnancies and 50 normal pregnancies.

**Intervention(s):**

None.

**Main Outcome Measure(s):**

The metabolic characteristics at the maternal-fetal interface in PCOS pregnancies and the underlying mechanisms.

**Results:**

A total of 79 metabolites in the placenta and 25 in umbilical cord serum showed significant differences between PCOS and normal pregnancies. The 10 most significant placental metabolites were identified through receiver operating characteristic analysis, 9 of which correlated significantly with maternal serum testosterone levels. Lasso regression analysis identified 4 key placental metabolite combinations: gamma-aminobutyric acid, proline, glycine, and isoleucine, achieving an area under the curve of 93.24%. In umbilical cord serum, 6 metabolites differed significantly between PCOS and normal pregnancies, with the highest area under the curve reaching 76.07%; 5 of these metabolites showed significant correlations with maternal serum testosterone levels. Nine differential metabolites were shared between the placenta and umbilical cord serum, which also shared metabolic pathways, including ABC transporters and aminoacyl-tRNA biosynthesis, potentially influencing maternal-fetal interactions.

**Conclusion:**

This study identifies the metabolomic profile and key pathways in maternal-fetal communication during PCOS pregnancies.

Polycystic ovary syndrome (PCOS) is the most common endocrinopathy among reproductive-age women, affecting approximately 11% to 13% of women worldwide (1). It is characterized by a spectrum of clinical manifestations including amenorrhea, hirsutism, acne, obesity, hypertension, hyperinsulinemia, insulin resistance, hyperandrogenism, and polycystic ovarian morphology (2). These symptoms not only affect reproductive health but also have broad systemic implications. Women with PCOS have a higher risk of impaired glucose tolerance, diabetes mellitus, depression, and anxiety, largely because of the underlying mechanisms (3). The multifaceted nature of PCOS underscores the need for a deeper understanding of its pathogenesis to improve therapeutic strategies and patient outcomes.

During normal pregnancy, significant metabolic adaptations occur to support fetal development (4). However, in women with PCOS, these processes are often disrupted, leading to metabolic dysregulation (5). Such disruptions can affect oocyte maturation, fertilization, and early embryonic development from abnormal metabolic levels in the follicles (6). Over time, these metabolic disturbances may subtly influence the course of pregnancy, increasing the risk of complications such as gestational diabetes, preeclampsia, preterm delivery, cesarean section, chorioamnionitis, maternal infections, and placental abruption (7, 8). For example, disrupted lipid metabolism during mid-pregnancy may adversely affect newborn birth weight (9). Additionally, newborns of women with PCOS are more likely to experience prolonged hospital stays and may be at increased risk of congenital anomalies (10). Despite the well-established association between PCOS pregnancies and adverse outcomes, different clinical studies with varying objectives often include heterogeneous patient populations, making it difficult to quantify and summarize the exact contribution of PCOS to adverse pregnancy outcomes and understand the specific mechanisms by which it exerts its effects.

Emerging evidence suggests that PCOS pregnancy may be closely related to abnormal placental development, which could contribute to placental dysfunction (11). This dysfunction is thought to be a key factor in the increased risk of miscarriage observed in pregnancies affected by PCOS (12). As we all know, the placenta plays a pivotal role in maternal-fetal interaction, serving as the primary interface where the exchange of nutrients, gases, and waste products occurs between mother and fetus (13). Consequently, disruptions in placental function can have far-reaching effects on pregnancy outcomes. Furthermore, the umbilical cord serum contains key information about this maternal-fetal communication. Analyzing both placental and umbilical cord serum samples allows for a more comprehensive understanding of the metabolic and endocrine changes in PCOS pregnancies, providing a more comprehensive view of the potential mechanisms behind adverse outcomes.

In this context, metabolomics, a powerful tool for the comprehensive analysis of metabolites offers a unique opportunity to uncover the biochemical alterations associated with PCOS in pregnancy despite existing studies identifying disrupted metabolism in maternal plasma of PCOS pregnancy (9). However, there is a paucity of research focusing on the metabolomic profiles of the placenta and umbilical cord serum in PCOS pregnancies. Understanding these profiles could provide important insights into how PCOS disrupts the normal metabolic pathways during pregnancy and its impact on maternal-fetal communication.

Therefore, to explore the metabolic features of maternal-fetal communication in uncomplicated PCOS pregnancies, we collected placenta and umbilical cord serum samples for gas chromatography-mass spectrometry (GC-MS) analysis to identify differential metabolites, key metabolic pathways, and their correlation with the clinical characteristics of PCOS.

## Materials and Methods

### Study Populations

To investigate the metabolic changes in the placenta and umbilical cord serum between normal pregnant women (control) and those with polycystic ovary syndrome (PCOS), a total of 95 pregnant women, including 45 with PCOS and 50 normal pregnant women, were recruited from the Chengdu Women's and Children's Central Hospital. This study was approved by the Ethics Committee of our hospital (2021105). Written informed consent was obtained from all participants before their inclusion in the study. All procedures involving human participants were conducted following the 1964 Declaration of Helsinki and the International Conference on Harmonisation Good Clinical Practice E6.

The diagnosis of PCOS was based on the 2003 Rotterdam criteria, requiring the presence of at least 2 of the following 3 symptoms before pregnancy: oligomenorrhea or amenorrhea (O), clinical and/or biochemical hyperandrogenism (H), and polycystic ovaries on ultrasound (P). The control group consisted of healthy women with no history of PCOS who received antenatal care at the same hospital.

To minimize the influence of external factors, we standardized the delivery method by selecting cesarean section, which is relatively consistent and less influenced by external variables. Participants were matched by gestational age, and body mass index (BMI) was kept similar across all subjects, ensuring a homogeneous sample that accurately reflects the metabolic profile of uncomplicated PCOS pregnancies. The exclusion criteria were: (1) pregnancies with complications; (2) use of medications or foods affecting hormones or metabolism within the past three months; and (3) presence of chronic medical disorders, including diabetes, cardiovascular disease, chronic kidney disease, collagen disorders, chronic hypertension, and metabolic diseases.

### Sample Collection

The maternal surface of the placentas (1 cm × 1 cm × 1 cm) was obtained immediately after delivery by elective cesarean section (within 30 minutes), avoiding macroscopic vessels as well as areas of calcification and infarction. After being rinsed with 0.9% saline, the placental tissues were rapidly frozen in liquid nitrogen and stored at −80 °C until metabolite extraction. Serum samples from cord blood were separated by centrifugation at 3000 rpm for 5 minutes at 4 °C and stored at −80 °C for later experiments (14). All samples were anonymized and collected, then matched with the baseline characteristics of the mothers and clinical data of the newborns.

### Sample Preparations for Serum and Tissue

The samples were all processed according to a standardized protocol (15, 16). A total of 300 μL of thawed serum was transferred to 1.5-mL tubes. A total of 20 μL of internal standard solution (10 mM d4-alanine and 10 mM d5-phenylalanine) were added to each tube. Then, 400 μL of cold methanol and 100 μL of 4 M sodium hydroxide were added to precipitate proteins. The mixture was vortexed and centrifuged at 1500 rpm for 15 minutes. The supernatant was collected for further derivatization.

Tissue samples (20.00 ± 0.50 mg) were placed into new tubes. Each sample was treated with a mixture of 1 M sodium hydroxide and methanol (1:1 v/v; 0.4 mL), two 3-mm tungsten carbide beads, and 10 μL of 10 mM D4-alanine. The samples were vortexed for 30 seconds, then homogenized using a TissueLyser II (QIAGEN, USA) at 30 Hz for 1 minute. After homogenization, the supernatant was separated by centrifugation at 12 000 rpm for 15 minutes at 4 °C and stored at 4 °C until further processing.

### Methyl Chloroformate Derivatization and GC-MS Analysis

For the stored supernatants, 34 μL of pyridine and 20 μL of methyl chloroformate (MCF) were first added, followed by 30 seconds of vigorous mixing using a vortex. Then, an additional 20 μL of MCF was added and mixed for another 30 seconds. Next, 200 μL of chloroform and 400 μL of sodium bicarbonate (50 mM) were added, and the mixture was vigorously mixed for 10 seconds. The mixture was then centrifuged at 2000 rpm for 10 minutes to separate the aqueous layer from the chloroform layer. After centrifugation, the aqueous layer was carefully removed and the remaining chloroform extract was dehydrated by the addition of sodium sulfate (∼0.3 g). Finally, the mixture was transferred to an amber glass GC-MS vial. Negative controls were prepared by subjecting an empty microcentrifuge tube to the identical processing steps as the samples. The volatile compounds were then separated using a ZB-1701 GC capillary column (30 m × 250 μm id × 0.15 μm with a 5 m guard column, manufactured by Phenomenex, CA, USA) and detected by GC-MS (Agilent 7890B-5977A) with electron impact ionization, employing electron emission at 70 eV. The GC-MS parameters were set according to the procedures described in previous research (17). The GC-MS inlet was heated to 290 °C, and the sample was introduced using the pulsed splitless mode at a flow rate of 1 mL/min for the helium carrier gas. The temperature was maintained at 280 °C, 230 °C, and 150 °C for the auxiliary, MS quadrupole, and MS source, respectively. The mass range was scanned between 30 μm to 550 μm, with a scan speed of 1.563 μ/s, and the MS detector was activated after 5.5 minutes (18).

### Metabolite Identification and Data Mining

Automated Mass Spectral Deconvolution & Identification System software was used for metabolite deconvolution and identification. Metabolites were identified by comparing their mass fragmentation patterns (relative intensity of mass spectra to the most abundant ion) and GC retention times, within 0.5-minute bins, against our in-house MS library, which was constructed using chemical standards. The MassOmics R-based software was used to extract the relative concentrations of the metabolites by using the peak height of the most abundant reference ion mass. To enhance quantitative robustness and minimize both instrumental and human variability, the relative concentrations of the identified compounds were normalized using internal standards (D4-alanine and D5-phenylalanine) and adjusted for the weight of placental tissue samples. The absolute concentrations of the targeted metabolites were determined using calibration curves derived from the corresponding chemical standards (19).

### Hormone Assay

Blood of pregnant women before delivery was taken from the elbow vein and centrifuged at 3000*g* for 10 minutes. Following centrifugation, the serum was promptly frozen at a temperature of −80 °C for subsequent hormonal testing. The concentration of testosterone was determined using an ELISA kit (Cloud-Clone Corp Cat# CEA458Ge, RRID: AB_2928143). The assay was conducted according to the kit's specifications, and the results were analyzed with a spectrophotometer (Bio Teck, Highland Park, USA) at a wavelength of 450 nanometers.

### Mendelian Randomization

A 2-sample Mendelian Randomization approach (20) was used to preliminarily explore the causal relationships between isoleucine (ebi-a-GCST90092843, met-d-Ile), proline (ebi-a-GCST90026014, met-a-355), glycine (ebi-a-GCST90092820, met-d-Gly, met-a-468, ebi-a-GCST90026013), leucine (ebi-a-GCST90092891, met-d-Leu, met-c-897, met-a-306), valine (ebi-a-GCST90092995, met-d-Val, met-c-940), and phenylalanine (ebi-a-GCST90092936, met-d-Phe, met-c-919) with PCOS (PCOS ID: ebi-a-GCST90044902). Exposure and outcome data were derived from independent samples, and the inverse variance-weighted method was solely applied to estimate the causal effects of these metabolites on PCOS.

### Statistical Analysis

Given the sample size of this study, the Shapiro-Wilk test was applied to assess the normality of all quantitative data. For data that followed a normal distribution, an unpaired Student *t*-test was used; for data that did not follow a normal distribution, the Mann-Whitney U test (rank-sum test) was applied. Qualitative data were analyzed using the chi-squared test.

The false discovery rates were assessed using the q-value function in the R program to account for multiple comparisons. Variables with a *P* value < .05 and a corresponding q-value (false discovery rate) < .05 were considered statistically significant. Significant metabolites were visually depicted in heat maps using the ggplot2 and ComplexHeatmap packages in R software (version 4.3.1).

The diagnostic ability was determined by the receiver operating characteristic (ROC) curve, with the area under the curve (AUC) commonly used to evaluate the predictability of the classifier, where a higher AUC value indicates better performance.

Feature selection for placental metabolites was conducted using Least Absolute Shrinkage and Selection Operator (Lasso) regression, implemented with the glmnet package in R software (version 4.3.1). The optimal value of lambda was determined through cross-validation.

Correlations between metabolites and the clinical characteristics were analyzed using Pearson's correlation coefficient for normally distributed data, and Spearman's rank correlation for nonnormally distributed data. Statistical significance was determined with a *P* value < .05.

Metabolic pathways were identified through KEGG metabolic pathways, and chord plots connecting metabolites with their associated metabolic pathways were created using the GOplot package in R software (version 4.3.1) (21).

## Results

### Clinical Characteristics

**Figure 1.**
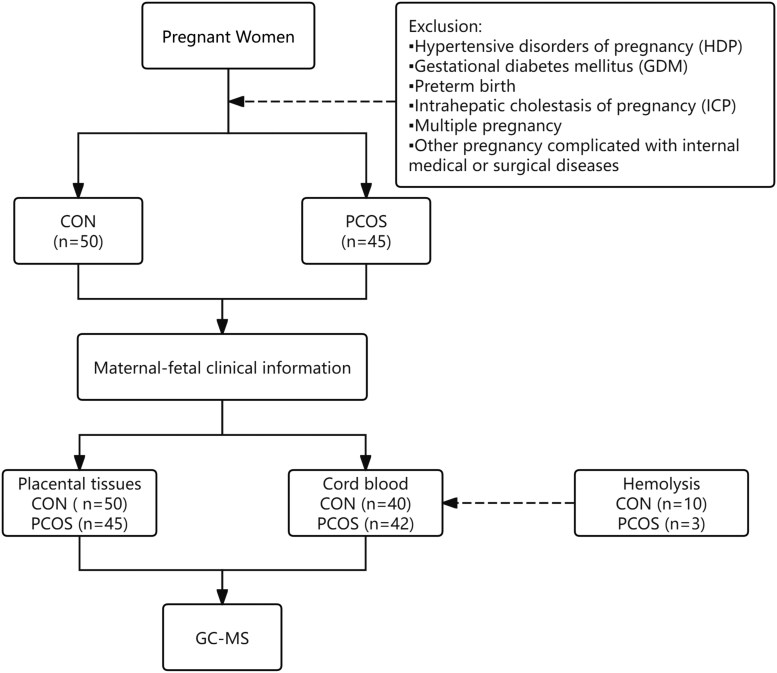
Flowchart of this study. Abbreviations: CON, control (normal pregnant women); GC-MS, gas chromatography-mass spectrometry; PCOS, pregnancies with polycystic ovary syndrome (PCOS).

The clinical characteristics of the participants are detailed in Table 1. There are no significant differences between the control and PCOS pregnancy groups in terms of maternal age, height, weight, prepregnancy BMI, gestational weight gain (GWG), BMI at delivery, and gestational weeks (delivery). In addition, no significant difference were observed in newborn gender or birth weight. The primary difference lies in maternal testosterone levels, which are significantly higher in the PCOS pregnancy group, as measured by ELISA (*P* < .001). The reliability of ELISA measurements was further confirmed by LC-MS analysis (Supplementary Fig. S1) (22), which showed a strong positive correlation (*R* = 0.9995, *P* < 2.2 × 10⁻¹⁶). The overall design of the study is depicted in Fig. 1.

**Table 1.** Clinical data of the participants

Variables	Total (n = 95)	Control group (n = 50)	PCOS pregnancy group (n = 45)	Statistic	*P*
Maternal age (year), M (Q₁, Q₃)	30.00 (26.00, 32.00)	29.00 (26.00, 32.00)	30.00 (26.00, 32.00)	*Z* = −0.16	.873
Maternal height (cm), mean ± SD	158.82 ± 4.96	158.66 ± 4.86	159.00 ± 5.12	t = −0.33	.741
Maternal weight at delivery (kg), M (Q₁, Q₃)	68.00 (64.00, 72.50)	68.00 (64.00, 72.00)	68.00 (64.00, 73.00)	*Z* = −0.05	.961
Maternal prepregnancy weight (kg), M (Q₁, Q₃)	55.75 (52.50, 60.00)	55.00 (52.50, 59.00)	56.00 (52.50, 60.00)	*Z* = −0.56	.575
Gestational weight gain (kg), M (Q₁, Q₃)	12.00 (10.00, 15.00)	12.50 (10.00, 15.00)	12.00 (10.00, 15.00)	*Z* = −0.40	.689
Maternal prepregnancy BMI (kg/m^2^), M (Q₁, Q₃)	22.31 (20.70, 23.45)	21.88 (20.58, 23.92)	22.43 (21.80, 23.23)	*Z* = −0.59	.552
Maternal BMI at delivery (kg/m^2^), M (Q₁, Q₃)	27.05 (25.73, 28.75)	26.64 (25.53, 28.98)	27.06 (26.03, 28.58)	*Z* = −0.11	.913
Newborn weight (g), M (Q₁, Q₃)	3280.00 (3065.00, 3540.00)	3265.00 (3087.50, 3495.00)	3300.00 (3050.00, 3590.00)	Z = −0.26	.797
Gestational age (week), M (Q₁, Q₃)	39.00 (38.43, 39.43)	39.00 (38.43, 39.43)	39.00 (38.29, 39.29)	Z = −0.55	.583
Newborn female, n (%)	47 (49.47)	23 (46.00)	24 (53.33)	χ²=0.51	.475
Maternal testosterone levels (pg/mL) M (Q₁, Q₃)	1626.75 (1280.90, 2251.28)	1328.55 (1132.51, 1605.43)	2295.54 (1834.29, 2734.75)	Z = −6.52	<.001

Abbreviations: BMI, body mass index; M, median; Q₁, 1st quartile, Q₃, 3rd quartile; *Z*, Mann-Whitney test.

### Metabolomic Profiling of Placenta and Umbilical Cord Serum in PCOS Pregnancies

**Figure 2.**
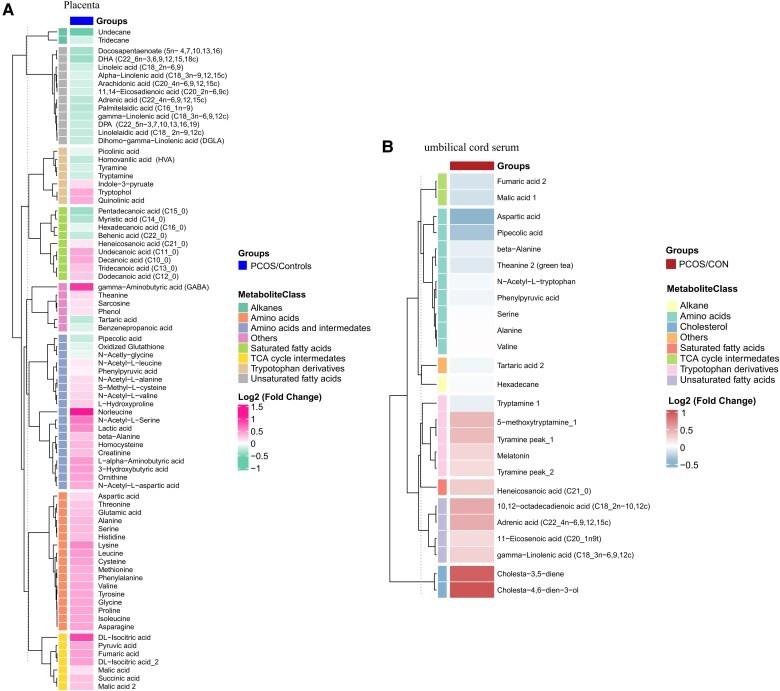
Differential metabolites in placenta and umbilical cord serum. Heatmaps display differentially expressed metabolites in placental tissue (A) and umbilical cord serum (B) grouped by metabolic class, with color gradients representing log2-fold changes between PCOS and controls. PCOS, pregnancies with polycystic ovary syndrome (PCOS).

Partial least-squares projection to latent structures analysis showed distinct metabolomic profiles in the placenta (Supplementary Fig. S2A) (22) and umbilical cord serum (Supplementary Fig. S2B) (22) of patients with PCOS compared to control group. Using in-house MCF mass spectral library, we identified 138 differential metabolites in the placenta and 144 differential metabolites in the umbilical cord serum. Among these, 79 significant metabolites in the placenta were statistically significant (*P* < .05, q-value < 0.05), including 26 downregulated and 53 upregulated metabolites (Supplementary Fig. S2C) (22). These metabolites exhibited notable changes in unsaturated fatty acids (such as docosapentaenoate, DHA, and linoleic acid), amino acids (including aspartic acid, threonine, and glutamic acid), and TCA cycle intermediates (Fig. 2A). Additionally, 25 significant metabolites were observed in the umbilical cord serum (*P* < .05, q-value < 0.05), with 13 downregulated and 12 upregulated (Supplementary Fig. S2D) (22). These findings highlight prominent changes in amino acids (such as aspartic acid, β-alanine, and serine), tryptophan derivatives (such as tryptamine and melatonin), and fatty acids (including gamma-linolenic acid and 10,12-octadecadienoic acid) (Fig. 2B).

### Identification of Key Metabolites of Placenta and Umbilical Cord Serum in PCOS Pregnancies

**Figure 3.**
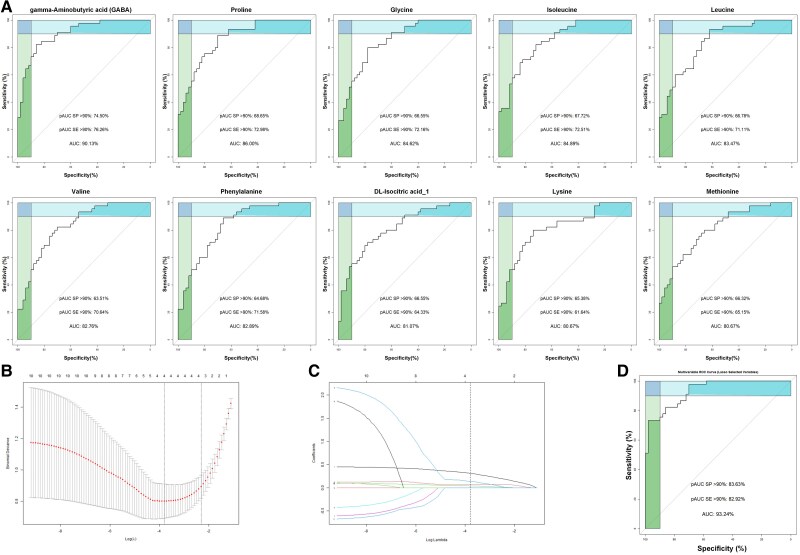
Screening of key metabolites in placenta: (A) ROC curves illustrating the discriminative power of individual metabolites for distinguishing PCOS pregnancies. Higher AUC values indicate stronger differentiation capabilities. (B) Scatter plots showing the positive correlation (R > 0) between specific metabolites and maternal testosterone levels. Significant correlations are marked with *P* < .05. (C) The changes of coefficients with lambda parameters. (D) ROC curve of the combined model using multiple metabolites to enhance the differentiation of PCOS pregnancies characteristics. Abrreviations: AUC, area under the curve; pAUC SE, partial area under the curve for sensitivity; pAUC SP, partial area under the curve for specificity; PCOS, polycystic ovary syndrome; R, Pearson correlation coefficient; ROC, receiver operating characteristic.

The ROC curve analysis of the significant metabolites identified in the placenta found 10 metabolites with an AUC above 80% for discriminating PCOS pregnancy from controls. Nine amino acids (proline, glycine, isoleucine, leucine, valine, phenylalanine, DL-isocitric acid_1, lysine, methionine), 1 tryptophan derivative (gamma-aminobutyric acid [GABA]) (Fig. 3A, Supplementary Table S1 (22)).

After lasso feature selection, 4 metabolites were screened out, namely GABA, proline, glycine, and isoleucine (Fig. 3B-3C, Table 2). By combining these 4 metabolites into a multivariate ROC curve model, the generated AUC was 93.24%, indicating that they have the potential to distinguish PCOS pregnancy from normal pregnancy (Fig. 3D).

**Table 2.** The corresponding coefficients of each variable under lambda

Variables	Coefficient
(Intercept)	−7.6344
Gamma-aminobutyric acid	0.3108
Proline	0.0665
Glycine	0.0394
Isoleucine	0.1380

Besides, there are 6 metabolites with ROC AUC exceeding 70% in umbilical cord serum, namely cholesta-3,5-diene, cholesta-4,6-dien-3-ol, melatonin, dopamine, tyramine peak_2, and histidine. The highest one is cholesta-3,5-diene (76.07%) (Supplementary Fig. S3, Supplementary Table S2) (22).

### Correlations Between Key Metabolites and Biochemical Markers in PCOS Pregnancy

**Figure 4.**
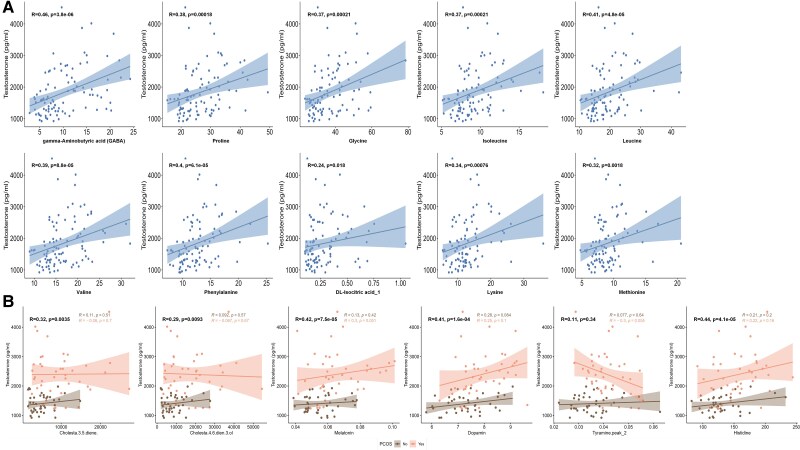
The association of key metabolites in placenta and umbilical cord serum with maternal testosterone levels. (A) Scatter plots showing the positive correlation (R > 0) between specific metabolites in placenta and maternal testosterone levels. Significant correlations are marked with *P* < .05. (B) Scatter plots showing the positive correlation (R > 0) between specific metabolites in umbilical cord serum and maternal testosterone levels. Significant correlations are marked with *P* < .05. The choice of Pearson or Spearman correlation depends on the normality of the data. Different styles of curves, shadows, and scatter points represent different groups. Abrreviations: AUC, area under the curve; pAUC SE, partial area under the curve for sensitivity; pAUC SP, partial area under the curve for specificity; PCOS, polycystic ovary syndrome; R, Pearson correlation coefficient; ROC, receiver operating characteristic.

Overall, the key placenta metabolites showed significant positive correlations with testosterone levels (Fig. 4A). Specifically, GABA (*R* = 0.46, *P* = 3.8 × 10⁻⁶), proline (*R* = 0.38, *P* = .00018), glycine (*R* = 0.37, *P* = .00021), isoleucine (*R* = 0.37, *P* = .00021), leucine (*R* = 0.41, *P* = 4.8 × 10⁻5), valine (*R* = 0.39, *P* = 8.8 × 10⁻5), phenylalanine (*R* = 0.4, *P* = 6.1 × 10⁻5), DL-isocitric acid_1 (*R* = 0.24, *P* = .018), lysine (*R* = 0.34, *P* = .00076), and methionine (*R* = 0.32, *P* = .0018) were all significantly correlated with testosterone levels of maternal serum.

However, further subgroup analysis revealed that the correlation between placental metabolites and testosterone levels in maternal serum was not significant in either the PCOS pregnancy group or the control group (Supplementary Fig. S4) (22). This suggests that the disease phenotype may counteract this association.

In umbilical cord serum, although 5 metabolites initially showed a positive correlation with testosterone levels in maternal serum, this significance was lost on subgroup analysis (Fig. 4B). Additionally, there was no significant correlation between key placental (Supplementary Fig. S5) (22) and umbilical cord serum metabolites (Supplementary Fig. S6) (22) and the birth weight of the newborns.

### Metabolite Feature and PCOS Causal Relationship Verification

In addition, Mendelian randomization analysis revealed that the key metabolites do not have a causal relationship with PCOS itself (Supplementary Table S3) (22), further proving that they alone represent the characteristics of PCOS pregnancy.

### Association of Identical Metabolites in Placental and Umbilical Cord Serum With Biochemical Markers in Pregnancies Complicated by PCOS

**Figure 5.**
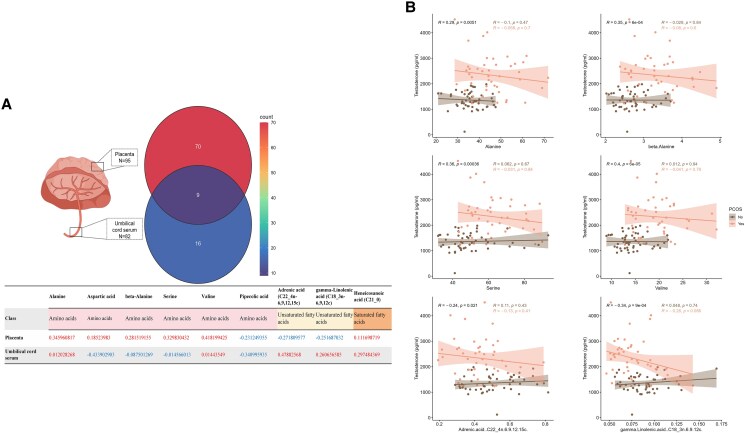
Shared metabolites between placenta and umbilical cord serum metabolites and their association with maternal testosterone levels. (A) Venn diagram showing shared metabolites between placenta and umbilical cord serum. The table highlights significantly upregulated (red) and downregulated (blue) metabolites in each tissue. (B) Scatter plots illustrating correlations between shared metabolites in the placenta and maternal testosterone levels. Correlation coefficients (R) represent positive or negative correlations, with significance indicated by *P* < .05. The choice of Pearson or Spearman correlation depends on the normality of the data. Different styles of curves, shadows, and scatter points represent different groups. R, correlation coefficient.

Nine identical metabolites were detected in both the placenta and umbilical cord serum, comprising 6 amino acids (alanine, aspartic acid, beta-alanine, serine, valine, and pipecolic acid), 2 unsaturated fatty acids (adrenic acid [C22: 4n-6,9,12,15c] and gamma-linolenic acid [C18: 3n-6,9,12c]), and 1 saturated fatty acid (heneicosanoic acid [C21: 0]). Among these, alanine, valine, and heneicosanoic acid (C21: 0) were upregulated in both tissues, whereas pipecolic acid was downregulated in both. The other metabolites exhibited opposite trends between the placenta and umbilical cord serum (Fig. 5A).

Despite the presence of identical metabolites, no significant correlation was observed between their levels in the placenta and umbilical cord serum (Supplementary Fig. S7) (22). However, 6 shared metabolites in the placenta—alanine, beta-alanine, serine, valine, adrenic acid (C22_4n-6,9,12,15c), and gamma-linolenic acid (C18_3n-6,9,12c)—were significantly correlated with maternal testosterone levels (Fig. 5B). In contrast, 1 shared metabolite in the umbilical cord serum, pipecolic acid, showed a strong correlation with maternal testosterone (Supplementary Fig. S8) (22). Notably, these differences were not significant in subgroup analyses. Additionally, 1 shared metabolite in the placenta, adrenic acid (C22_4n-6,9,12,15c), was significantly correlated with newborn weight in the control group (*P* = .016) (Supplementary Fig. S9) (22), whereas no correlations were observed between other shared metabolites, either in the placenta or umbilical cord serum (Supplementary Fig. S10) (22), and newborn weight.

### Predicting Metabolic Pathway Activity in Placenta and Umbilical Cord Serum

**Figure 6.**
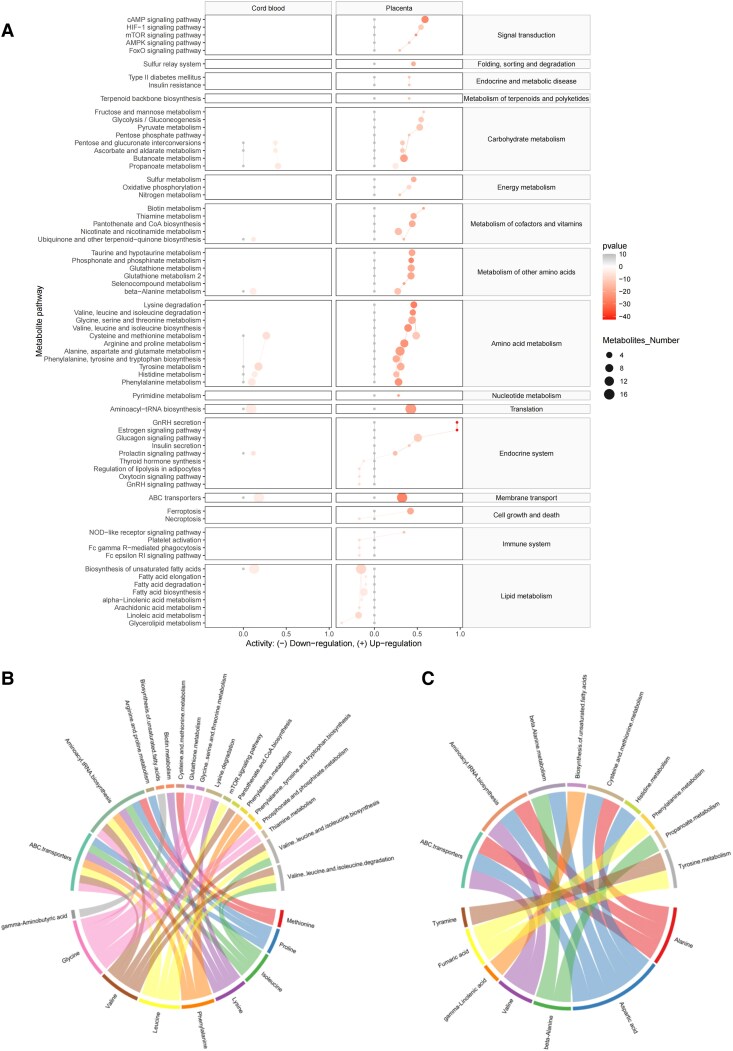
Shared metabolic pathways between placental and umbilical cord serum and corresponding metabolites. (A) Heatmap illustrates the metabolic pathways of umbilical cord serum (left) and placenta (right), with variations in shading intensity representing the *P* values and size indicating the number of metabolites involved. The chord diagram depicts the participation of key metabolites of placenta (B) and significant metabolites of umbilical cord serum (C) in various significant metabolic pathways.

In PCOS pregnancy, metabolic pathways in the placenta and umbilical cord serum exhibit significant alterations (Fig. 6A). Specifically, the placental metabolome in PCOS pregnancy is predominantly impacted in pathways related to signal transduction and energy metabolism including “signal transduction,” “amino acid metabolism,” “lipid metabolism,” and “membrane transport.” In contrast, the umbilical cord serum exhibits fewer altered pathways, which are almost identical to those in the placenta. Notably, the “biosynthesis of unsaturated fatty acids” is upregulated in the umbilical cord serum but downregulated in the placenta. The shared metabolic pathways between the placenta and umbilical cord serum, including “carbohydrate metabolism,” “metabolism of cofactors and vitamins,” “amino acid metabolism,” “metabolism of other amino acids,” “translation,” “endocrine system,” “membrane transport,” and “lipid metabolism,” may facilitate the transmission of maternal signals, influencing fetal growth and development.

In Fig. 6B, we show the roles of 9 significant placental metabolites related to testosterone (*P* < .05) and with ROC curve AUC > 0.8 in metabolic pathways. Metabolic pathway enrichment analysis showed that proline, isoleucine, lysine, phenylalanine, leucine, valine, and glycine are involved in the ABC transporters and aminoacyl-tRNA biosynthesis pathways. Proline also participates in arginine metabolism, lysine in biotin metabolism and degradation, and methionine in methionine metabolism. Glycine is linked to multiple pathways, including glutathione and thiamine metabolism. Leucine, valine, and isoleucine are involved in branched-chain amino acid metabolism, with leucine also linked to mTOR signaling. Phenylalanine is involved in its metabolism and biosynthesis of related amino acids. GABA participates in unsaturated fatty acid biosynthesis. A chord diagram of the complete placental metabolites and the corresponding enriched pathways is provided in Supplementary Fig. S11 (22). Furthermore, Fig. 6C shows how the differential metabolites in umbilical cord serum participate in the enrichment pathways.

### Exploring Shared Metabolic Pathways Between Placental and Umbilical Cord Serum

**Figure 7.**
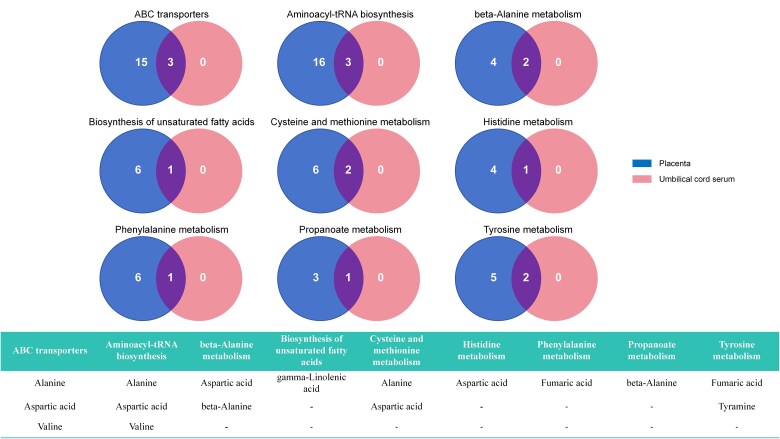
The intersection of metabolites corresponding to shared pathways in placenta and umbilical cord serum.

To reveal the metabolic characteristics of the placenta and umbilical cord serum in shared metabolic pathways. In Fig. 7, we analyzed the differential metabolites present in both tissues. Although these pathways exist in both the placenta and umbilical cord serum, the expression of differential metabolites varies between these tissues. Nevertheless, all differential metabolites detected in the umbilical cord serum have corresponding manifestations in the placenta.

Among the key findings, valine, a significantly upregulated metabolite in the placenta, also exhibits elevated levels in the umbilical cord serum. Valine plays an active role in both the ABC transporter and aminoacyl-tRNA biosynthesis pathways in both tissues. Furthermore, alanine and aspartic acid are important shared metabolites involved in several pathways, participating in the ABC transporter, aminoacyl-tRNA biosynthesis, and cysteine and methionine metabolism pathways. Despite being present in both samples, the concentrations and functional roles of these metabolites differ, likely reflecting the unique metabolic demands and functions of the placenta and umbilical cord serum.

## Discussion

Epidemiological evidences suggest that PCOS pregnancy is associated with a higher risk of adverse outcomes, potentially because of altered placental function (23, 24). However, the underlying biological mechanisms are not fully understood. In this study, we performed a comparative analysis of the placenta and umbilical cord serum metabolomes between PCOS-affected pregnancies and healthy controls. After feature selection, several metabolites were identified that can effectively distinguish PCOS pregnancies from normal pregnancies. Many of these metabolites have been described as characteristic markers in the circulating metabolism of the PCOS population and showed similar trends (25). Key metabolic pathways involved include membrane transport, translation, lipid metabolism, and amino acid metabolism. These findings offer novel insights into the pathophysiological mechanisms underlying PCOS-related pregnancy complications.

A notable observation in our study was that there were no significant differences between the PCOS pregnancy group and the control group in terms of maternal weight, prepregnancy BMI, GWG, delivery BMI, and neonatal birth weight. This contrasts with previous studies suggesting that women with PCOS are more prone to obesity and abnormal birth weight outcomes resulting from insulin resistance and lipid metabolism issues (26). However, there are also causal association studies that confirm PCOS does not affect the birth weight of offspring (27, 28). Furthermore, maternal BMI and GWG have been confirmed to directly influence fetal birth weight (29). Thus, these differences may stem from variations in the study population, as we matched for BMI and excluded women with comorbidities like obesity or gestational diabetes. This ensured a milder, purer form of PCOS, allowing for a clearer understanding of its true impact on maternal-fetal communication. Therefore, the impact of PCOS alone on newborn weight may not be as significant as previously thought. Future studies should explore if PCOS indirectly influences newborn weight by worsening other pregnancy complications. Additionally, differences in lifestyle from ethnic and regional dietary habits may also contribute to the observed discrepancies. Tracking participants' food diaries could help clarify potential associations.

Proline metabolism and its role in oxidative stress have been studied in PCOS (30) but its impact during PCOS pregnancy remains underexplored. Valine and phenylalanine have also been described as metabolic characteristics of PCOS (31). Although pregnancy can reduce elevated valine levels caused by PCOS, they remain higher than normal pregnancy levels (32). Consistent with previous studies, phenylalanine levels in PCOS are higher than those in normal populations (33), and this change is also reflected in the placenta. Glycine levels, often reduced in nonpregnant PCOS patients, were significantly increased in PCOS pregnancies, possibly because of changes in metabolic status, placental function, and inflammation (34). Leucine and isoleucine levels were elevated during PCOS pregnancy, indicating that pregnancy may exacerbate metabolic disturbances similar to those observed in obese PCOS (35, 36). DL-isocitrate, an intermediate in the tricarboxylic acid cycle (37), was also increased, suggesting a link to mitochondrial function and oxidative stress (38). Elevated lysine levels may reflect increased inflammation, lipid metabolism abnormalities, or hormonal imbalances (39, 40). Methionine levels were abnormally elevated, possibly because of changes in DNA methylation that could be passed on to offspring (41). Finally, PCOS women have higher levels of tryptophan (36). GABA, as an inhibitory neurotransmitter, is a derivative of tryptophan and is involved in the regulation of the nervous system, and GABA concentrations are elevated in the serum (42) and cerebrospinal fluid (43, 44) of PCOS women. Previous studies have also revealed that PCOS may be associated with an imbalance in GABA-mediated hypothalamic regulation (45). The neural regulatory mechanism in PCOS pregnancy has not yet been studied. The significant differences in GABA found in the placenta in this study further suggest that there may be a potential regulatory mechanism for the placenta-brain axis in PCOS pregnancy.

Mendelian randomization analysis showed no significant causal relationship between the differential metabolites and PCOS itself (20), indicating these metabolites reflect unique characteristics of PCOS pregnancy. Circulating testosterone levels remain high in PCOS pregnancies at term (46), which was also verified in this study. The 10 most significantly different placental metabolites were positively correlated with PCOS, consistent with previous studies linking hyperandrogenism to elevated leucine and isoleucine levels (34). Further research is needed to explore whether other metabolites may indirectly relate to maternal testosterone levels by affecting protein synthesis or other pathways. There was no significant correlation observed between the differential metabolites and birth weight. This may be because the influence of metabolites on offspring is not limited to birth weight alone, but may also involve more subtle changes, such as abnormalities in other unknown indicators or alterations in neurodevelopment.

We found 9 identical metabolites in the metabolites of placenta and umbilical cord serum, which are likely to mediate maternal-fetal communication and affect maternal-fetal outcomes in both directions. Among the metabolites, alanine, valine, and heneicosanoic acid (C21: 0) exhibited an increasing trend, whereas pipecolic acid showed a decreasing trend. Valine, as a key metabolite (47), demonstrated a similar trend in both placenta and cord blood, suggesting its role in energy exchange between mother and fetus. Elevated alanine suggests the need for further research on its impact on fetal metabolism. Heneicosanoic acid, though less studied, may influence maternal-fetal lipid metabolism. Some metabolites in both the placenta and umbilical cord serum were linked to maternal testosterone levels, but their effect on birth weight seems to depend on the tissue. Further research is needed to understand how these metabolites influence maternal-fetal interaction in PCOS pregnancies.

We focused on shared metabolic pathways between the placenta and umbilical cord serum because their alterations in PCOS pregnancy may contribute to adverse maternal and fetal outcomes. Specifically, changes in signal transduction and energy metabolism pathways may disrupt the fetal energy supply (48), increasing the risk of fetal growth restriction (49) or macrosomia (50). Alterations in carbohydrate and lipid metabolism suggest that PCOS pregnancy may disrupt glucose and lipid balance between the mother and fetus, potentially leading to adverse outcomes such as gestational diabetes (51), abnormal fetal fat deposition (52), and premature labor (8). Abnormalities in amino acid metabolic pathways, particularly when observed in both the placenta and umbilical cord serum, could significantly impact fetal growth (53), immune function (54), and nervous system development (55). Additionally, the opposite regulation of unsaturated fatty acid biosynthesis in the placenta and umbilical cord serum reflects the dynamic balance of lipid metabolism between mother and fetus. This imbalance may impair the fetus's acquisition of essential fatty acids, thereby affecting the brain (56, 57) and retinal development (58) and increasing the risk of premature labor or low birth weight (59). Changes in cofactor and vitamin metabolism could affect the placenta's and fetus's ability to absorb and use key nutrients, potentially leading to decreased placental function and an increased risk of miscarriage (60), fetal malformations (61), or gestational hypertension (62). Finally, abnormalities in the endocrine system and membrane transport pathways may directly impact the exchange of hormones and nutrients between the mother and fetus (63), resulting in abnormal fetal growth and development.

To identify key target metabolites and pathways in PCOS pregnancy, we further analyzed the differential metabolites associated with the shared metabolic pathways in the placenta and umbilical cord serum. It was found that ABC transporters and aminoacyl-tRNA biosynthesis played significant roles in these shared pathways. Among the corresponding metabolites, valine, a key shared metabolite between the placenta and umbilical cord serum, emerged as particularly important. This suggests that valine may facilitate maternal-fetal interaction in PCOS pregnancy through its involvement in the ABC transporters and aminoacyl-tRNA biosynthesis pathways. ABC transporters are a crucial class of transmembrane proteins responsible for transporting a variety of molecules—including metabolites, drugs, and lipids—across cell membranes (64). In the placenta, they play a central role in the exchange of substances between mother and fetus (65). As a branched-chain amino acid, valine may further influence the amino acid supply and energy exchange between mother and fetus via the ABC transporter pathways. Additionally, aminoacyl-tRNA synthetases are responsible for attaching amino acids to tRNA, forming aminoacyl-tRNA, a necessary step in protein synthesis (66). This metabolic pathway is enriched in people with PCOS peripheral serum (67). The synthesis and metabolism of valine, being an essential amino acid, directly impacts protein production (68). Overall, the metabolic pathways involving valine, such as ABC transporters and aminoacyl-tRNA biosynthesis, may play a critical role in the metabolic regulation of pregnant women with PCOS. These pathways could affect maternal-fetal material exchange, potentially influencing both short-term and long-term outcomes for the mother and fetus.

The limitations of this study include the limited external validity due to its single-center design and the lack of longitudinal data across different stages of pregnancy. Additionally, although some metabolites associated with PCOS pregnancy were identified, the specificity of these biomarkers needs further validation through animal studies or clinical trials. Further research with larger, more diverse samples, combined with longitudinal data and mechanistic studies, is necessary to better understand the metabolic changes in PCOS pregnancy and their impact on maternal and fetal health.

In conclusion, this study deeply explored the metabolic changes in the placenta and umbilical cord serum in PCOS pregnancy, explored the close relationship between differential metabolites and clinical characteristics, and revealed the interaction between mother and fetus in multiple key metabolic pathways related to signal transduction, amino acid metabolism, and lipid metabolism. The findings of this study emphasize the importance of metabolic regulation in PCOS pregnancy and provide potential targets for metabolic intervention in PCOS pregnancy in the future.

## Data Availability

The supplementary figures and tables supporting the findings of this study are available at Zenodo (https://doi.org/10.5281/zenodo.14598100); further inquiries can be directed to the corresponding author.

## Abbreviations

AUC: area under the curve
BMI: body mass index
GABA: gamma-aminobutyric acid
GC-MS: gas chromatography-mass spectrometry
GWG: gestational weight gain
MCF: methyl chloroformate
PCOS: polycystic ovary syndrome
ROC: receiver operating characteristic
