# Correction to: “Metabolic Profiles of Pregnancy With Polycystic Ovary Syndrome: Insights into Maternal-Fetal Metabolic Communication”

**DOI:** 10.1210/clinem/dgaf189

**Published:** 2025-03-27

In the above-named article by Ge H, Huang D, Tan L, Luo D, Zhou L, Liu H, Zhang Y, Liu D, Wu X, Wang L, Xiong L, Yang Y, Han T-L, He C, and Qi H (*J Clin Endo Metab*. 2025; doi: 10.1210/clinem/dgaf057), there was an error in the legend for Figure 3.

In the originally published article, in the legend for Figure 3, the description of panel C read “Finding a lambda value that minimizes error while maintaining model simplicity,” and the description of panel D read “The changes of coefficients with lambda parameters.” This is incorrect because images were repositioned during the revision stage and the legend was not edited to reflect the changes. Therefore, legends originally listed under “D” and “E” are now attributed to panels C and D, respectively, and the original description for panel C has been deleted. In the corrected article, the description of panel C now reads “The changes of coefficients with lambda parameters,” and the description of panel D now reads “ROC curve of the combined model using multiple metabolites to enhance the differentiation of PCOS pregnancies characteristics.”

The article has been corrected online.

doi: 10.1210/clinem/dgaf057

